# Familiarity mediates the relationship between emotional arousal and pleasure during music listening

**DOI:** 10.3389/fnhum.2013.00534

**Published:** 2013-09-05

**Authors:** Iris van den Bosch, Valorie N. Salimpoor, Robert J. Zatorre

**Affiliations:** ^1^Neuroscience and Cognition, Graduate School of Life Sciences, Utrecht UniversityUtrecht, Netherlands; ^2^Montreal Neurological Institute, McGill UniversityMontreal, QC, Canada; ^3^International Laboratory for Brain Music and Sound Research (BRAMS), Montreal, QC, Canada

**Keywords:** music, familiarity, pleasure, emotional arousal, psychophysiology, EDA

## Abstract

Emotional arousal appears to be a major contributing factor to the pleasure that listeners experience in response to music. Accordingly, a strong positive correlation between self-reported pleasure and electrodermal activity (EDA), an objective indicator of emotional arousal, has been demonstrated when individuals listen to familiar music. However, it is not yet known to what extent familiarity contributes to this relationship. In particular, as listening to familiar music involves expectations and predictions over time based on veridical knowledge of the piece, it could be that such memory factors plays a major role. Here, we tested such a contribution by using musical stimuli entirely unfamiliar to listeners. In a second experiment we repeated the novel music to experimentally establish a sense of familiarity. We aimed to determine whether (1) pleasure and emotional arousal would continue to correlate when listeners have no explicit knowledge of how the tones will unfold, and (2) whether this could be enhanced by experimentally-induced familiarity. In the first experiment, we presented 33 listeners with 70 unfamiliar musical excerpts in two sessions. There was no relationship between the degree of experienced pleasure and emotional arousal as measured by EDA. In the second experiment, 7 participants listened to 35 unfamiliar excerpts over two sessions separated by 30 min. Repeated exposure significantly increased EDA, even though individuals did not explicitly recall having heard all the pieces before. Furthermore, increases in self-reported familiarity significantly enhanced experienced pleasure and there was a general, though not significant, increase in EDA. These results suggest that some level of expectation and predictability mediated by prior exposure to a given piece of music play an important role in the experience of emotional arousal in response to music.

Music has been around for as long as history can tell us, and is present in all known cultures around the world (McDermott, 2008). Moreover, it continues to evolve: new pieces of music are created on a daily basis and new music styles arise regularly. Furthermore, we know that listening to music is one of the most pleasurable experiences to humans (Dubé and Le Bel, 2003). But how do we start liking new music? To what extent do we experience pleasure the first time we hear a novel piece of music? How does that experience change as we hear it more often?

The pleasurable aspects of music listening are thought to arise from the ability of music to convey, induce, and modulate a wide range of emotions. There are many sources of emotion in music (Juslin and Västfjäll, 2008) but one influential theory has been that these emotions are caused by expectancies and anticipation of events that occur in the music (Meyer, 1956; Huron, 2006). Music is the organization of tones over time, and listeners assemble implicit, generalized knowledge of musical rules and regularities via mere exposure to music of a given genre (Tillmann, 2005). Through this abstract knowledge, listeners create expectancies, and depending on whether these expectations are violated or confirmed, listeners can experience tension and suspense or relaxation (Meyer, 1956; Huron, 2006). This implicit understanding of the rules of music has been labeled as structural knowledge by Bharucha (1987). The resulting moments of anticipation and resolution are believed to be one basis for emotions in response to music. However, when listeners are familiar with a specific piece of music, anticipation may also arise because they know explicitly that a particular event is followed by another; this aspect has been termed veridical knowledge by Bharucha (1987). These two types of knowledge are not mutually exclusive, since the second form of anticipation evolves from the first, and overall anticipation is likely to be a combination of both. In the present study we use novel and unfamiliar music in order to remove the influence of veridical knowledge and examine emotional responses based only on structural knowledge. This approach can allow us to disentangle the subtle differences that exist between these two sources of anticipation and better understand the emotional implications of anticipation within the music itself.

To investigate emotional experiences during music listening, we need a way to measure emotions objectively. Although emotions are subjective states, they are accompanied by physiological changes in the body (Sequeira et al., 2009). More specifically, emotional arousal is characterized by increased physiological activity mediated by the autonomic nervous system. Within the domain of music emotion research, physiological measures such as electrodermal activity (EDA), heart rate, respiration, and body temperature have been frequently used as correlates of emotional arousal (Krumhansl, 1997; Gomez and Danuser, 2004, 2007; Rickard, 2004; Salimpoor et al., 2009). Amongst these, EDA is generally a preferred measure as it is highly sensitive and under strict control of the sympathetic nervous system (and is therefore largely involuntary; Cacioppo et al., 2007; Sequeira et al., 2009). Furthermore, a relationship between EDA as indicator of emotional arousal and experienced pleasure in response to music has previously been demonstrated (Khalfa et al., 2002; Rickard, 2004; Grewe et al., 2009; Salimpoor et al., 2009).

EDA consists of two main components: skin conductance level (SCL) corresponding to the tonic sympathetic nervous discharging, and skin conductance responses (SCR) correlated with phasic discharges of sympathetic innervations (for a detailed description, see Cacioppo et al., 2007; Sequeira et al., 2009). Both SCL and SCR have demonstrated a relationship with subjective ratings of pleasure and arousal. SCL measures in response to emotional music show significantly greater and more positive percent change relative to baseline than relaxing and arousing music (Rickard, 2004). Furthermore, SCL is significantly higher when listeners consider the music pleasurable, rather than neutral, and both SCL and SCR increase significantly when listeners experience “chills” (i.e., moments of peak pleasure) in response to music (Grewe et al., 2009; Salimpoor et al., 2009). Although EDA has been demonstrated to be a reliable indicator of emotional arousal, it should be mentioned that it is less accurate with respect to detecting the valence dimension of emotions. Since we are only interested in the pleasurable aspect of music accompanied by positive valence, this does not pose a problem here. A strong relationship thus exists between pleasure, emotional arousal and EDA.

Despite the potential importance of familiarity, the studies mentioned above have not taken it directly into account (Rickard, 2004; Grewe et al., 2009); other studies have used music excerpts selected by the subjects as emotional or pleasurable (Rickard, 2004; Salimpoor et al., 2009) which were highly familiar and predictable whereas the neutral control music was unfamiliar. In line with the aforementioned theory concerning anticipation (Meyer, 1956; Huron, 2006), one could therefore argue that the increases in EDA reported in these prior studies may have been influenced by familiarity because subjects were anticipating specific moments in the music known to induce emotions, instead of, or in addition to, experiencing pleasure purely induced by the structure of the music itself. To clarify this issue and to investigate if a similar relationship between pleasure and emotional arousal as measured by EDA can be found when we hear a piece of music for the first time, novel music should be introduced. Finding novel music pieces that elicit a pleasurable response can be an extremely difficult task due to the wide variety of musical preferences. We overcame this problem by first selecting a group of individuals with highly similar tastes in music, after extensive pilot testing. We then selected new music specifically for this group of people, based on their musical preferences. Although not all new musical pieces in the genre will necessarily be experienced as pleasurable by everyone, this procedure increases the likelihood that many of the selections will be liked by most people. Furthermore, different degrees of pleasure across subjects and the resulting variability in pleasure allows for examination of the underlying changes in emotional arousal.

In addition to examining the relationship between pleasure and emotional arousal when listening to a piece of music for the first time, the use of novel music also allows for examination of the effect of exposure on emotional arousal. It is well-known that liking for unfamiliar items typically increases as a function of exposure (Zajonc, 1968; Bornstein, 1989). This effect has also been demonstrated within the musical domain (Heingartner and Hall, 1974; Peretz et al., 1998). Moreover, this effect remains even when listeners have no explicit memory for the previously encountered stimulus (Zajonc, 1980; Szpunar et al., 2004; Schellenberg et al., 2008). It should be noted that this effect can be seen with as few as two repetitions over a short time period, and that, subsequently the effect inverts as a result of satiation (Szpunar et al., 2004; Schellenberg et al., 2008), but only when listeners explicitly recognize the musical stimuli. A similar relation between exposure, musical complexity and liking has been shown, associating increased exposure with decreased complexity (Berlyne, 1970; Orr and Ohlsson, 2001). Furthermore, it has recently been shown that higher familiarity with musical chords increased the experience of consonance and consequently pleasantness ratings (McLachlan et al., 2013). It has also been demonstrated that exposure to unfamiliar note sequences enhances melodic expectancy judgments, even in the absence of explicit recognition (Thompson et al., 2000). The effects of exposure and familiarity on emotional arousal are however still unknown. According to the theory that musically induced emotions arise in part from anticipation (Meyer, 1956; Huron, 2006), we may infer that enhanced expectations are related to the enhanced liking judgments, which in turn might be related to an increase in emotional arousal.

The aim of the present study is to investigate how we start liking an unfamiliar, novel piece of music. This question is examined in two ways. First, in experiment 1, we will examine if the previously established relationship between the pleasurable experiences of music listening and emotional arousal as measured by EDA continues to exist when unfamiliar music is used. Second, in experiment 2, we will examine if exposure has an effect on emotional arousal by exposing listeners twice to novel pieces of music. We hypothesize that when novel pieces are experienced as pleasurable, emotional arousal will be enhanced. Furthermore, we hypothesize that novel pieces heard for the second time will be judged more pleasurable, and will be accompanied by enhanced emotional arousal.

## Materials and methods

### Subjects

Over 250 individuals responded to advertisements posted around the university campus and sent to various university and community email lists. To ensure that respondents listen to new music, the advertisement recruited individuals buying a lot of new music and stated that reimbursement would consist of a $10 iTunes gift certificate card. Further screening to assess musical preferences was conducted via email by inquiring about their favorite music genre and favorite music artists. When respondents' favorite music genres included “Indie,” “Rock,” or “Electronic” and their favorite artists included indie-performers and had overlap with the majority of other selected respondents, they were invited to participate in the study. A total of 60 subjects, 28 males and 32 females with a mean age of 25 in the range of 18–50 participated in the experiment; musical experience varied widely but formal musical training was not assessed systematically. All subjects were believed to be healthy and free from any psychological or neurological disorder and gave written informed consent before participating in the study. Ethical consent was approved by the McGill University Research Ethics Board.

### Stimuli

To increase ecological validity and to obey copyright issues, the same 30 s that are used as samples of the complete songs in the iTunes Store (http://www.apple.com/itunes/what-is/store.html) were recorded using Audacity software (http://audacity.sourceforge.net/). A pool of potential stimuli was collected based upon a series of criteria: (1) resemblance to the music listed by the subjects as their favorite music (i.e., based upon the musical preferences of the subjects themselves); (2) recent release date (i.e., to minimize the possibility for subjects to recognize the music); (3) representative of the indie genre (e.g., indie pop, indie rock, indie electronic, etc.). Of this collection, 70 stimuli were selected which were presumed to elicit a wide range of pleasurable responses. In previous studies we did not use music that contained lyrics in order to ensure that emotional arousal can be elicited by music alone, and found that this was indeed the case (Blood and Zatorre, 2001; Salimpoor et al., 2009, 2011). As such, for the current study we did not exclude music with lyrics in order to increase ecological validity of the stimuli. Wavefiles were normalized to a maximum amplitude difference of ±3 dB to minimize the differences in sound levels between the different music fragments.

### Procedure

Subjects were tested individually in one session lasting approximately 1.5 h in a sound proof room at the Montreal Neurological Institute. After giving informed consent, subjects were provided with an outline of the experimental procedure and were given instructions. Subjects were then fitted with the psychophysiological equipment to record EDA, two 11 mm Ag/AgCl dry electrodes secured with Velcro straps placed on the distal phalanges of the index and ring finger of the non-dominant hand. The task consisted of 70 trials. On each trial subjects first listened to a 30 s music fragment and were then asked to give ratings on an 11-point scale assessing their experienced pleasure (from −5: hated it to +5: loved it), arousal (from 0: calm to 10: extremely excited), and familiarity (from 0: never heard before to 10: know very well). Ratings were given by moving a cursor on a computer screen to the appropriate position through button presses with the dominant hand. The importance of rating pleasure and arousal as actually felt while listening to each music piece was emphasized before the start of the experiment. Instructions for familiarity ratings emphasized that 0 meant they never heard it before, 1 meant they heard it once, 2 meant they heard it twice, etc., with 10 meaning they knew it very well. Subjects were also instructed to minimize all body movements during the entire experiment so as not to disturb the physiological measurements. Data was collected at a rate of 256 Hz (Biograph Infiniti Software, Thought Technology Ltd.). Music was played through headphones (Sennheiser) and volume was adjusted to a pleasant listening level (as indicated by the subjects themselves). Distractions were minimized and subjects were asked to comfortably position themselves in an armchair, upon which they were acquainted with the task and its response methods on 3 practice trials. The actual task was divided into two runs of 35 trials each with a break in between. During the break, electrodes were removed and subjects were asked to complete questionnaires assessing their musical training, listening habits, and musical preferences. Before each run, EDA data were collected over a 2-min silent relaxation period, which served as baseline to account for inter-individual differences in physiological activity. Upon completion, subjects were reimbursed for their participation, and asked not to divulge any of the details of the experiment to other potential subjects.

### Experiment 1

Experiment 1A was designed to investigate the relationships between the pleasurable experiences of listening to novel music and emotional arousal as measured by EDA; only musical excerpts rated as entirely unfamiliar were used. Experiment 1B was designed to investigate the difference in this possible relationship between listening to both novel and familiar music, by including excerpts that had higher familiarity ratings than those used in Experiment 1A. A subgroup of 33 subjects who met all criteria (see below) were included in this experiment. The stimuli, task, and procedures were as described above, with each of the 70 trials of the task in this experiment consisting of different music fragments, the order of presentation counterbalanced among subjects.

### Experiment 2

Experiment 2 was designed to examine the effect of familiarity on emotional arousal via stimulus repetition. A different subgroup of 7 individuals participated in this experiment which was similar to the first experiment, with the only difference that during the second run, all items from the first run were repeated a second time. In other words, during the second run, participants heard the same 35 music excerpts they heard in the first run, randomized in a different order, rather than entirely novel music excerpts as in Experiment 1. The repeated music excerpts were counterbalanced among subjects so that the 70 music pieces were presented (approximately) an equal number of times. While participants were not told that the musical selections would be repeated during the second run, their explicit awareness of whether each of the excerpts had been heard before was assessed when they rated the familiarity of each piece during the second run. The purpose of this experiment was to examine the changes in emotional arousal associated with increased familiarity to novel musical stimuli.

## Analyses

To prepare the EDA data for analysis, signal filtering was performed with a low-pass filter of 0.6 Hz to remove motion-induced noise and artifacts. Over long recording times, the EDA signal exhibits a slow downward drift due to charge accumulation at the electrode-skin junction, causing a linear decrease of conductance. The EDA signal was therefore detrended using a piecewise linear regression model. Furthermore, EDA can vary widely between different subjects or within the same subject in different psychological states [for more detailed information see Cacioppo et al. (2007)]. A particular subject might for instance have a tonic SCL around 10 μS (microsiemens) ranging within 2 μS, whereas another subject might have a SCL around 5 μS ranging within 1 μS. Thus, to be able to directly compare EDA differences amongst participants, two methods of quantification were applied. First, to account for inter-individual differences in physiological activity, the mean SCL value of the data collected during baseline was subtracted from the SCL data collected during the actual task. Second, range correction, a procedure recommended by Lykken and Venables (1971; Lykken et al., 1966), was performed to account for the inter-individual differences in variance. This procedure involves computing the possible range for each individual subject and then expressing the subject's momentary value in terms of this range. More specifically, by determining a subject's minimum and maximum SCL, the subject's present SCL can be expressed as a proportion of its individualized range according to the formula (SCL – SCLmin)/(SCLmax – SCLmin). This procedure reduces error variance and increases the power of statistical tests on the data set. For each subject, the resulting EDA signal was subsequently segmented into 70 epochs of 30 s each, corresponding to the times when subjects were listening to music excerpts. The mean SCL value and the number of SCR's (nSCR) were computed for each excerpt. Multiple One-Way repeated measures analysis of variances (ANOVA) were performed. For experiment 1A, Pleasure, Arousal, and Run were used as the independent variables and SCL and nSCR values as the dependent variables. For experiment 1B and 2, Familiarity and Exposure were respectively added as independent variables and, in addition to SCL and nSCR, both Pleasure and Arousal were used as dependent variables. Greenhouse–Geisser corrections were carried out when appropriate.

### Experiment 1

Only subjects who showed enough variance in their pleasure ratings (i.e., at least 5 items in every pleasure category from 0 to 5) were included in the analyses. Furthermore, data from one subject had to be excluded due to technical difficulties. The analyses presented here include data collected from 33 subjects (18 males, 22 females, mean age of 23 ranging from 18 to 37). For the purpose of experiment 1A, 535 out of the 2314 music fragments that were rated negatively on the valence dimension (i.e., excerpts rated lower than 0) and/or high on familiarity (i.e., excerpts rated higher than 0) were excluded from analyses. For experiment 1B, only music excerpts rated negatively on the valence dimension were excluded from the analyses (444 out of 2314). Thus, we included for this analysis the excerpts rated as having some degree of familiarity, which were excluded in the analysis of experiment 1A. The within-subject factors for experiment 1A were Pleasure (six levels, from “neutral” to “loved it”), Arousal (eleven levels, from “extremely calm” to “extremely aroused”) and Run (two levels, Run 1 and Run 2). For experiment 1B, the within-subject factor Familiarity (three levels, Unfamiliar: rating 0, Somewhat Familiar: rating 1–5, and Very familiar: rating 6–10), derived from the subjective familiarity ratings, was added.

### Experiment 2

Experiment 2 consisted of a different group of 7 subjects (4 males, 3 females, mean age of 28 in the range of 19–50). Within this dataset, music excerpts rated negatively on the valence dimension and high on familiarity (i.e., excerpts rated higher than 5) were again excluded from the analyses. The additional within-subject factor for experiment 2 was Exposure (two levels, Exposure 1 and Exposure 2), to test the effect of exposure via stimulus repetition during the second run of the experiment.

## Results

### Experiment 1

#### Experiment 1a

Participants' ratings are displayed in Table 1. On average, subjects rated 55 out of the 70 music fragments positive on valence showing that we had been able to select music pieces that conform to subjects' musical preferences. Mean SCL and nSCR values for Pleasure and Arousal are displayed in Tables 2, 3. No significant effect of Pleasure was found on SCL [*F*_(3.12, 81.08)_ = 0.63, *p* = 0.61] or nSCR [*F*_(2.82, 73.26)_ = 0.48, *p* = 0.69] and no significant effect of Arousal was found [F_(1, 1)_ = 1.95, *p* = 0.40] for SCL and [*F*_(1, 1)_ = 1.11, *p* = 0.44 for nSCR]. Also, no significant correlations were found between pleasure and SCL (*r* = 0.033, *p* = 0.66) or nSCR (*r* = 0.002, *p* = 0.98) or between arousal and SCL (*r* = 0.036, *p* = 0.57) or nSCR (*r* = 0.093, *p* = 0.14). Furthermore, no effect of Run on SCL or nSCR was found [*F*_(1, 32)_ = 0.01, *p* = 0.95 and *F*_(1, 32)_ = 0.41, *p* = 0.53].

**Table 1:** **Mean music excerpts (SD) rated in positive pleasure categories**.

**Pleasure 0**	**Pleasure 1**	**Pleasure 2**	**Pleasure 3**	**Pleasure 4**	**Pleasure 5**
8.79	9.82	11.73	11.88	8.31	4.44
(4.77)	(4.85)	(4.95)	(5.05)	(4.18)	(3.7)

**Table 2:** **Mean SCL in μS (SD) and nSCR (SD) values for positive pleasure categories**.

	**Pleasure 0**	**Pleasure 1**	**Pleasure 2**	**Pleasure 3**	**Pleasure 4**	**Pleasure 5**
SCL	0.31 (0.02)	0.31 (0.01)	0.31 (0.02)	0.31 (0.02)	0.32 (0.02)	0.34 (0.03)
nSCR	4.29 (0.42)	4.27 (0.31)	4.32 (0.36)	4.32 (0.34)	4.37 (0.34)	4.69 (0.34)

**Table 3:** **Mean SCL in μS (SD) and nSCR (SD) values for arousal categories**.

	**Arousal 0**	**Arousal 1**	**Arousal 2**	**Arousal 3**	**Arousal 4**	**Arousal 5**
SCL	0.32 (0.02)	0.23 (0.02)	0.33 (0.08)	0.26 (0.01)	0.25 (0.06)	0.22 (0.02)
nSCR	4.34 (0.23)	6.43 (1.57)	4.61 (0.90)	5.42 (0.09)	3.69 (0.69)	3.79 (0.22)
	**Arousal 6**	**Arousal 7**	**Arousal 8**	**Arousal 9**	**Arousal 10**
SCL	0.28 (0.06)	0.29 (0.04)	0.29 (0.08)	0.27 (0.04)	0.44 (0.05)
nSCR	4.02 (0.62)	4.42 (0.33)	3.92 (0.09)	4.50 (0.17)	4.50 (1.50)

#### Experiment 1b

**Figure 1:**
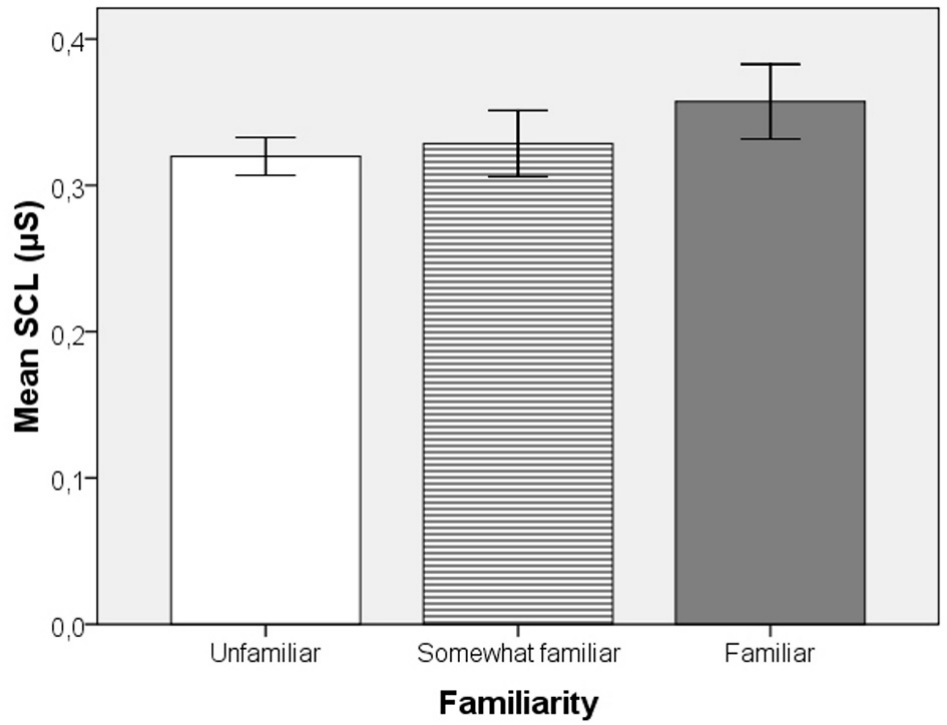
**Mean skin conductance level values as a function of familiarity from Experiment 1B.** The trend toward higher values with familiarity is not statistically significant.

When including both unfamiliar and familiar music excerpt to examine the role of familiarity in EDA, One-Way within-subject ANOVAs for SCL and nSCR with the within-subject factor Familiarity was conducted. No significant effect of Familiarity was found on SCL [*F*_(1.56, 39.06)_ = 1.73, *p* = 0.19] or nSCR [*F*_(1.59, 39.76)_ = 1.14, *p* = 0.32]. However, the slight trend toward an increase in SCL with higher familiarity, depicted in Figure 1, might be relevant; we therefore explored this potential relationship further via non-parametric tests.

Non-parametric Friedman's ANOVA did not reveal any significant effect of Familiarity on SCL [χ^2^_(2)_ = 3, *p* = 0.23] or nSCR [χ^2^_(2)_ = 1.46, *p* = 0.49]; yet a significant effect of Familiarity on Pleasure [χ^2^_(2)_ = 34.65, *p* < 0.001] and Arousal [χ^2^_(2)_ = 27.32, *p* < 0.001] was found. *Post-hoc* comparisons with Bonferroni correction revealed significant differences in pleasure ratings for all comparisons [Unfamiliar compared to Somewhat Familiar (*T* = 408, *r* = −0.51)], Somewhat Familiar compared to Very Familiar (*T* = 305, *r* = −0.47), and Unfamiliar compared to Very Familiar (*T* = 402, *r* = −0.56). *Post-hoc* comparisons also revealed significant differences in arousal ratings for all comparisons [Unfamiliar compared to Somewhat Familiar (*T* = 389, *r* = −0.45), Somewhat Familiar compared to Very Familiar (*T* = 309, *r* = −0.42), and Unfamiliar compared to Very Familiar (*T* = 392, *r* = −0.53)].

**Figure 2:**
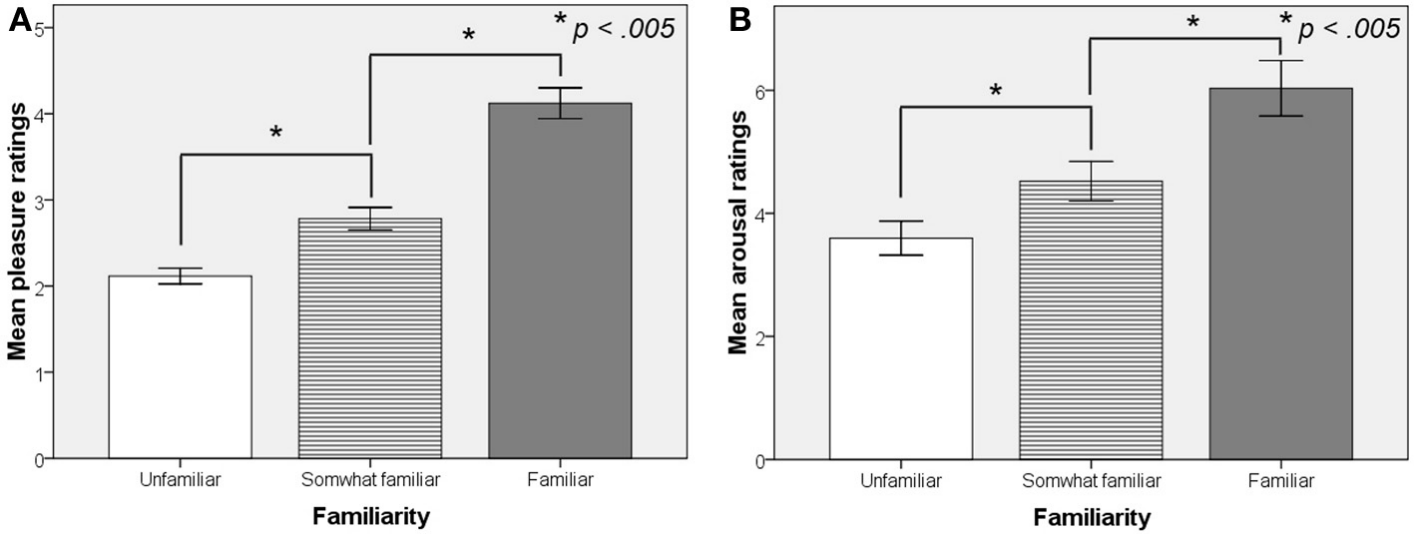
**Effect of Familiarity on mean pleasure ratings (A) and mean arousal ratings (B) from Experiment 1B.** All differences are statistically significant.

The effect of Familiarity on Pleasure and self-rated Arousal is displayed in Figure 2. Familiarity was revealed to have a significant positive effect on Pleasure [*F*_(1.52, 37.94)_ = 58.3, *p* < 0.001]. *Post-hoc* comparison with Bonferroni correction revealed significant differences in pleasure ratings for all levels of Familiarity (*p* < 0.005 in all comparisons). A significant positive effect of Familiarity was also found on arousal ratings [*F*_(1.27, 31.84)_ = 25.05, *p* < 0.001] with significant differences (*p* < 0.005 in all comparisons) for all levels of Familiarity as revealed by *post-hoc* comparison using Bonferroni correction.

#### Experiment 2

**Figure 3:**
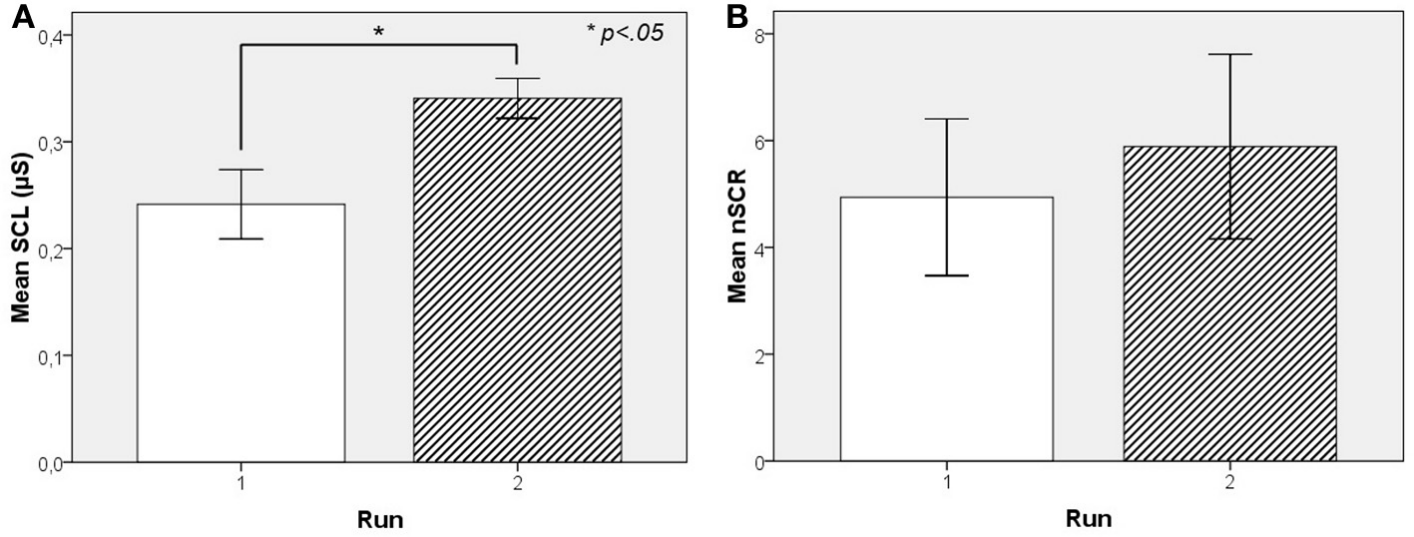
**Mean skin conductance level values as a function of exposure from Experiment 2.** Exposure (run 1 vs. run 2) to the same musical pieces results in significantly higher mean SCL values **(A)** but not nSCR values **(B)**.

The within-subject factor Exposure and its effect on SCL and nSCR was investigated with a One-Way within-subject ANOVA, revealing a significant positive effect of Exposure on SCL [*F*_(1, 6)_ = 11.78, *p* < 0.05] and on nSCR [*F*_(1, 6)_ = 5.72, *p* = 0.05] as shown in Figure 3. However, no effect of Exposure was found on Pleasure [*F*_(1, 6)_ = 0.19, *p* = 0.68] or Arousal [*F*_(1, 6)_ = 0.01, *p* = 0.93]. As was mentioned above, no effect of Run on SCL or nSCR was found in experiment 1 showing that the effect observed in experiment 2 is not an artifact related to the experimental design such that higher skin conductance occurs as a function of time.

**Figure 4:**
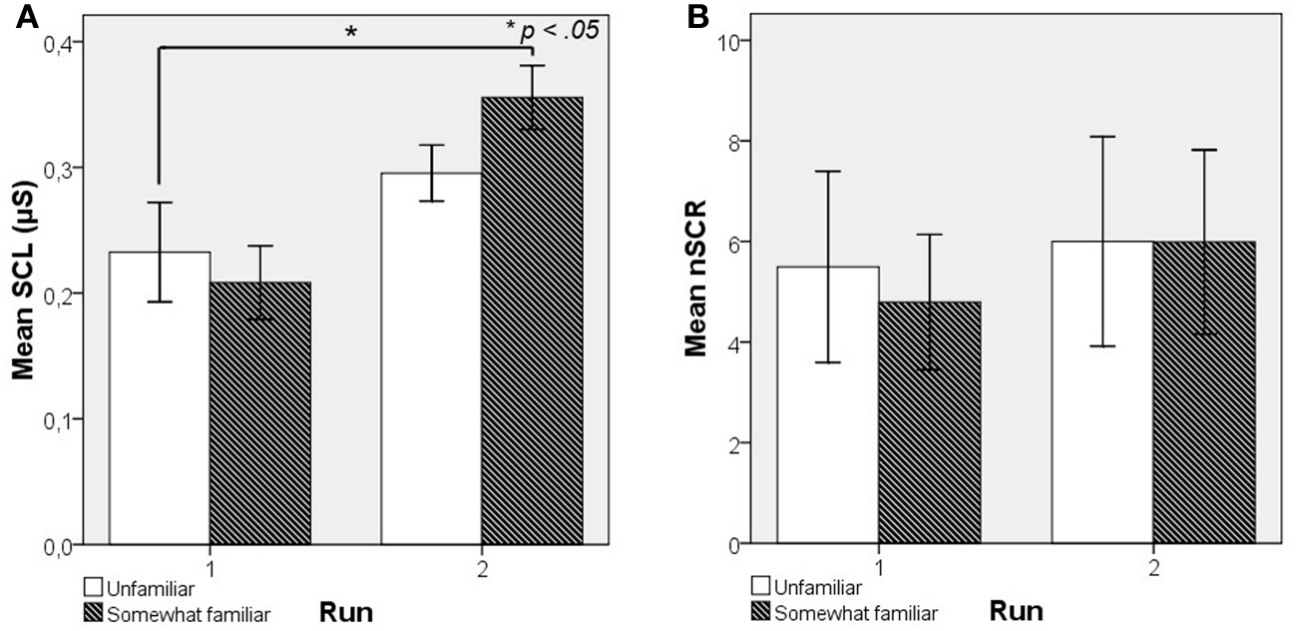
**Mean skin conductance level values as a function of self-rated familiarity from Experiment 2: SCL values (A) and mean nSCR values (B) grouped by familiarity**.

To see whether exposure to novel music excerpts also has an effect on EDA when subjects are not explicitly aware that they have heard an excerpt before, we grouped the music excerpts according to subjective familiarity ratings. Figure 4A shows that excerpts rated as Somewhat familiar during the second exposure in the second run have a significantly higher mean SCL [*F*_(1, 5)_ = 10.579, *p* < 0.05] than those during first exposure in the first run whereas excerpts still rated as Unfamiliar during the second exposure do not have a significantly higher mean SCL [*F*_(1, 6)_ = 2.762, *p* = 0.148]. Mean nSCR did not differ significantly between exposures for excerpts rated Unfamiliar [*F*_(1, 6)_ = 2.047, *p* = 0.202] or Somewhat familiar [*F*_(1, 5)_ = 4.594, *p* = 0.085] as shown in Figure 4B.

## Discussion

The results of this study show two findings. First, listening to novel music can elicit pleasurable experiences. However, within the context of unfamiliar music no relationship was found between the degree of experienced pleasure and emotional arousal as measured by EDA. Second, and more importantly, familiarity with music pieces influences emotional arousal as measured by EDA. More specifically, repeated exposure to novel music significantly increased SCL and significantly increased experienced pleasure.

It was mentioned in the introduction that previous studies have already shown a relationship between EDA and experienced pleasure in response to music (Khalfa et al., 2002; Rickard, 2004; Grewe et al., 2009; Salimpoor et al., 2009), though some of these studies used highly familiar and predictable music excerpts selected by the subjects as pleasurable whereas the neutral control music was unfamiliar. This difference in familiarity necessarily yields a difference in listeners' expectations about what will happen in the music, which is of central importance when comparing emotional responses to music. Emotional responses to music are thought to be linked to the confirmation or violation of expectations about music, as is stated in the influential theory on emotion and meaning in music (Meyer, 1956). Neurobiological evidence for this theory comes from a recent study showing a functional dissociation in dopaminergic activity in response to pleasurable experiences to familiar music (Salimpoor et al., 2011). Whereas the nucleus accumbens was more involved during the actual experience of a peak moment of pleasure, the caudate was more involved during anticipation of this peak moment of pleasure. The caudate is tightly connected to the dorsolateral prefrontal cortex and dorsal anterior cingulate cortex, (Haber and Knutson, 2010) regions known to be involved in the integration of knowledge from memory and ongoing events (Stuss and Knight). These findings indicate that expectations, predictions, and anticipation can result in dopamine release in this area, which can in turn lead to dopaminergic activity in the limbic area. Because dopamine is the main chemical involved in desires and addictive behaviors (Leyton, 2009), this anticipation may be viewed as a form of auditory craving resulting in emotional responses depending on the confirmation or violations of the expectations (Salimpoor et al., 2011). This idea fits as well with broader concepts of reward prediction that have been described in a large body of physiological literature (Zald and Zatorre, 2011).

However, expectations, predictions, and anticipation are partly dependent on familiarity. By using only unfamiliar novel music in our first experiment, we excluded the possibility that a difference in emotional arousal between pleasurable and neutral music was due to expectations based on explicit knowledge (i.e., memory representations) of the music excerpts themselves. As we did not find even a trend toward a relation between EDA and experienced pleasure with unfamiliar music, despite the relatively large subject sample, we conclude that knowledge of a specific music excerpt may contribute to expectations, which in turn influences emotional arousal. This idea is supported by the results of our second experiment, where an increase in exposure was found to significantly increase EDA. Although the sample size in Experiment 2 was much smaller than in Experiment 1, the effect was statistically significant, indicating that it must be relatively robust to be detected even with fewer subjects. We therefore suggest that familiarity may have an important mediating role when assessing the relationship between emotional arousal and pleasure in response to music. However, we cannot discount the possibility that under different testing circumstances, or a much larger test sample, EDA might be sensitive to pleasure or other aspects of emotion even for completely new music. A larger-scale study exploring other experimental conditions would no doubt be valuable in testing these conclusions.

Repeated exposure to unfamiliar music excerpts has previously been shown to positively influence melodic expectancy judgments (Thompson et al., 2000). This effect of exposure on expectancies was implicit, as subjects did not report explicit recognition of the music excerpts they had been exposed to. The results of our second experiment agree with these findings, and extend the effects of exposure to physiological arousal as measured by EDA, as demonstrated by the positive effect of Exposure on EDA. When subjects in the second run of the task were exposed to the same set of stimuli as in the first run, a significant increase in SCL was observed. However, this increase in SCL was not always accompanied by an increase in self-reported familiarity or pleasure. The absence of a significant increase in self-reported familiarity for the second run of the task illustrates that subjects were not, for the most part, explicitly recognizing the music excerpts as having been heard before. This may also explain why an effect was only found for SCL and not for nSCR. Tonic changes in SCL, such as those recorded over long-duration stimuli, differ from the phasic changes in the form of nSCR that occur 1–4 s after discrete events in long-duration stimuli (Miller and Shmavonian, 1965). nSCR may thus reflect the degree to which certain events within the stimuli stand out, concurrent with earlier findings of increased SCR when listener experience chills in response to music (Salimpoor et al., 2009). Without explicitly recognizing the music as heard before, the amount of events associated with nSCRs is likely to stay stable. Furthermore, the absence of an increase in self-reported familiarity is in accordance with the effect of exposure on expectancies, where even in the absence of explicit memory for previously encountered stimuli, exposure did increase the degree to which melodies conformed to expectations (Thompson et al., 2000). Together, these findings suggest that mere exposure may increase emotional arousal by increasing the listener's veridical and dynamic expectations.

The absence of an increase in experienced pleasure for the second run of the task does however not correspond with previous findings of the effects of exposure. As was mentioned in the introduction, it is well-known that exposure to novel music pieces increases the experienced pleasure (Heingartner and Hall, 1974; Peretz et al., 1998). Based on these well-documented effects of exposure on liking ratings we would expect to see an increase in self-reported pleasure, which perhaps might be more obvious under different testing conditions. However, we did observe the expected relation between the degree of familiarity and experienced pleasure, but only when familiarity for music excerpts was explicitly rated by the subjects themselves. Unlike the situation of mere exposure, self-reported familiarity automatically entails explicit recognition of the music excerpts. In this case, familiarity was found to correlate positively with subjective ratings of experienced pleasure and arousal. When a music excerpt was well-known, it was rated significantly higher on pleasure than a less known music excerpt, and this effect was also present when comparing completely novel excerpts with those that were just somewhat familiar (e.g., heard only once or twice). This observation may suggest that explicit expectations contribute more to experienced pleasure than implicit expectations, which is in accordance with the earlier mentioned studies by Salimpoor et al. (2009, 2011). In these studies, subjects may have been consciously anticipating their peak moments of pleasure in addition to implicit expectations, since they explicitly knew that a particular part of the music was coming up. The confirmation of these expectancies resulted in a significant increase in pleasure and EDA. Although a slight increase in EDA related with increased self-reported familiarity and pleasure was found in the present study, the lack of significance of this increase may be accounted for by the fact that it emerged from a small stimulus sample size. The music excerpts used to derive this result were selected to be novel to the subjects and as a result far more excerpts were rated unfamiliar than familiar (2215 vs. 99). The effect could therefore be stronger with higher numbers of familiar excerpts, a point which should be tested in future studies.

In sum, the present results suggest that some level of familiarity, expectation, and predictability may play an important role in the experience of emotional arousal and pleasure in response to music. These results are in accordance with findings of a recent study by McLachlan et al. (2013), who showed a significant role for familiarity in the experience of consonance and thus, pleasantness ratings of music. Nevertheless, we do not claim that familiarity and predictability are absolutely necessary for the experience of pleasure and arousal. For example, in addition to differences in emotional arousal across music excerpts, Blood and Zatorre (2001) and Salimpoor et al. (2009, 2011) have shown differences in psychophysiological and neural responses that correlated with emotional responses at different time points within pieces of music. These changes in emotional arousal and pleasure cannot be attributed to changes in familiarity since listeners' familiarity did not vary *within* the pieces of music. This finding clearly illustrates that familiarity should not be regarded as a necessary factor in the elicitation of emotional responses to music. Furthermore, schematic expectations are based on general musical rules or rules of a specific musical culture that are assimilated over a lifetime of listening experience. Listeners do not need to be explicitly familiar with a particular piece to predict what will happen based on these forms of expectations that can still result in emotional arousal and pleasure. This is exemplified by previously found relationships between EDA and pleasure in studies that employed experimenter-selected music and can be explained within the context of subjective appraisal processes. Lastly, extra-musical cues may also play a role in influencing a listeners' emotional response to novel music. For instance, knowing or hearing that a novel song is from an artist that we already like may prime us toward a more pleasurable response.

Subjective affective experiences are thought to consist of a complex interplay of two processes, an automatically occurring implicit appraisal process, often accompanied by physiological changes, and an explicit appraisal process which is a slower evaluation and relies more on individual factors (Scherer, 2004; Brattico and Jacobsen, 2009). In the musical domain, automatic implicit appraisal is a bottom-up process, largely related to the intrinsic structural aspects of the music itself, whereas explicit appraisal is a top-down, conscious evaluation process involving individual variables such as personal preference, motivation, past listening experiences, social attitude, etc. Studies with experimenter-selected music that revealed a relation between pleasure and EDA used musical stimuli that were a priori known to induce specific emotions. In other words, these studies used stimuli for which the emotional content had been previously assessed and validated. For instance, music pieces were extreme prototypes of different emotional categories inducing clearly distinct emotions (e.g., happy, sad, fearful, peaceful; Khalfa et al., 2002). These kinds of music excerpts generally exhibit specific structural aspects that give rise to certain emotions and are related to specific changes in physiological responses (Gomez and Danuser, 2007). Studies using these kinds of stimuli may therefore have been assessing this automatic appraisal process with the physiological changes that accompany them. The novel stimuli used in our study may have been more evaluated via the explicit conscious appraisal process, which may also explain why we were not able to find a relation between experienced pleasure and EDA in our first experiment.

A different interpretation follows from physiological theories of emotion, proposing that intense emotions are quantitatively different from mild emotions in that their intensity is determined by the individual's level of arousal (Schachter and Singer, 1962; Rickard, 2004). According to this view, intense pleasure can thus be distinguished from a neutral state by the degree of arousal. Perhaps not entirely coincidentally, one of the caveats of previous research has been that changes in EDA seemed to be more related to changes in arousal than to changes in valence (Bradley and Lang, 2000a,b; Khalfa et al., 2002; Sequeira et al., 2009). Significant differences in EDA were mostly found between high-arousing and low-arousing stimuli whereas fewer differences were found between negative-valence and positive-valence stimuli. In the present study we aimed to overcome this problem, as mentioned in the introduction, by excluding stimuli that were experienced as unpleasant. Presuming that a neutral state corresponds with low valence and low arousal while experienced pleasure corresponds with positive valence and high arousal, we would still be able to detect a difference. However, no significant relation between pleasure and EDA or between arousal and EDA was found.

Taken together, the results presented here suggest that increases in experienced pleasure in response to novel music pieces do not necessarily correspond with an increase in emotional arousal as measured by EDA. However, our results do show that familiarity may enhance expectations and predictability and provide evidence that these expectations and predictions play an important role in the elicitation of emotional and electrodermal responses. As such, we show that familiarity should be considered as an important factor when investigating emotional reactions and their physiological correlates. Caution should thus be exercised when this factor is not controlled. For instance, in previous research where pleasurable stimuli selected by the subjects were compared to stimuli selected by the experimenters, familiarity could have been a possible confounding, or at least contributing, factor in both pleasure ratings as well as in physiological responses. Familiarity with the pleasurable self-selected music may have enhanced emotional and physiological responses, resulting in larger differences between emotional and neutral music. Nonetheless, previous research has also been able to find differences in emotional and physiological responses within music stimuli for which listeners' familiarity was globally constant. This observation shows that the elicitation of emotions in response to music is a complex process, whose contributing factors and their integration are still poorly understood. Thus, to be able to draw firm conclusions on this topic with respect to familiarity, future research should investigate the role of familiarity and exposure on emotional and physiological responses more thoroughly.

The present study has some limitations, which should be considered in planning future studies on this topic. The effect of exposure that we report arose from a small sample size, and should therefore first be replicated with a larger population of subjects before making strong assumptions about its implications. We did not assess the role of musical training, which is an important topic for future research as well. Furthermore, while the effect of familiarity on liking ratings is well-known, future research should try to determine the contribution of self-reported familiarity on physiological responses when experienced pleasure is held constant, which could be done by pairing stimuli that differ in their degree of familiarity but elicit the same emotional reactions. The current study used music with lyrics to implement an ecologically valid sample of music most people listen to; future studies may want to separately examine the independent contribution of lyrics to familiarity and pleasurable responses to music. Results of such studies will increase our understanding of the role of familiarity in affective responses to musical stimuli.

### Conflict of interest statement

The authors declare that the research was conducted in the absence of any commercial or financial relationships that could be construed as a potential conflict of interest.
